# A dosimetric comparison of stereotactic body radiation therapy techniques for lung cancer: robotic versus conventional linac‐based systems

**DOI:** 10.1120/jacmp.v11i3.3223

**Published:** 2010-06-29

**Authors:** Chuxiong Ding, Cheng‐Hui Chang, Joshua Haslam, Robert Timmerman, Timothy Solberg

**Affiliations:** ^1^ Department of Radiation Oncology University of Texas Southwestern Medical Center 5801 Forest Park Road Dallas TX 75390 USA

**Keywords:** stereotactic body radiation therapy (SBRT), 4D CT, CyberKnife, Synchrony Respiratory Tracking System

## Abstract

The aim of this study is to compare the dosimetric characteristics of robotic and conventional linac‐based SBRT techniques for lung cancer, and to provide planning guidance for each modality. Eight patients who received linac‐based SBRT were retrospectively included in this study. A dose of 60 Gy given in three fractions was prescribed to each target. The Synchrony Respiratory Tracking System and a 4D dose calculation methodology were used for CyberKnife and linac‐based SBRT, respectively, to minimize respiratory impact on dose calculation. Identical image and contour sets were used for both modalities. While both modalities can provide satisfactory target dose coverage, the dose to GTV was more heterogeneous for CyberKnife than for linac planning/delivery in all cases. The dose to 1000 cc lung was well below institutional constraints for both modalities. In the high dose region, the lung dose depended on tumor size, and was similar between both modalities. In the low dose region, however, the quality of CyberKnife plans was dependent on tumor location. With anteriorly‐located tumors, the CyberKnife may deliver less dose to normal lung than linac techniques. Conversely, for posteriorly‐located tumors, CyberKnife delivery may result in higher doses to normal lung. In all cases studied, more monitor units were required for CyberKnife delivery for given prescription. Both conventional linacs and CyberKnife provide acceptable target dose coverage while sparing normal tissues. The results of this study provide a general guideline for patient and treatment modality selection based on dosimetric, tumor and normal tissue sparing considerations.

PACS numbers: 87.53.Ly, 87.55.dk.

## I. INTRODUCTION

Lung cancer is the leading cause of cancer death in United States among both men and women.^(^ ^1^ ^)^ Radiation therapy remains an essential modality of treatment, generally in an adjuvant setting with surgery and/or chemotherapy.^(^ ^2^ ^)^ In recent years, stereotactic body radiation therapy (SBRT) has been introduced as a primary treatment for lung cancer, with promising results in excellent tumor control rates as well as limited toxicities to normal tissue.^(^ ^3^ ^)^ Since the essential characteristic of SBRT is to deliver an ablative dose in a few fractions, accurate and reproducible patient setup and compensation for tumor motion are more critical than in conventional radiation therapy. The planning target volume (PTV), which is delineated from gross tumor volume (GTV), must be as small as reasonably possible in respect to normal tissue tolerances, without compromising target dose coverage.

A number of linac‐based approaches have been described for delivery of SBRT in lung cancer patients.^(^ ^3^ ^–^ ^4^ ^)^ Localization and interfraction setup uncertainties can be significantly reduced through the application of various image‐guided methodologies including orthogonal kV X‐ray imaging and cone‐beam computed topography (CBCT). Although management of intrafraction tumor motion due to patient respiration remains a challenge in SBRT treatment of lung cancer, several techniques are available to help reduce the impact of intrafraction uncertainties. The most straightforward method for compensating for tumor motion is to create internal target volume (ITV) by adding an appropriate margin to the GTV. However, this method may irradiate excessive normal tissue and/or miss the target by not considering the unique tumor motion in individual patients. 4D CT can provide temporal information on target and organ motion based on respiration and can be useful to in defining patient‐specific ITVs.^(^ ^5^ ^)^ It has been reported that composite images based on 4D CT scans, such as maximum intensity projection (MIP), minimum intensity projection and average intensity (AVG), are effective for the assessment of tumor mobility and ITV delineation.^(^ ^6^ ^,^ ^7^ ^)^ Respiratory gating^(^ ^8^ ^)^ is an potential method for reducing ITVs, with the tradeoff of increased treatment times. Methods such as breath hold, abdominal compression, and active breathing control^(^ ^9^ ^–^ ^11^ ^)^ have also been shown to be effective in reducing tumor motion during treatment. These methods can reduce the volume of normal tissue irradiated, but can be limited by such factors as patient comfort and compliance. In the future, tracking the tumor directly may prove to be an effective technique. Tumor tracking techniques, based on fluoroscopy or portal imaging or combined techniques,^(^ ^12^ ^–^ ^14^ ^)^ have been described. Although these methods are still under development and not in wide‐spread use in most institutions, they may help to further reduce the dose to the critical tissue.

CyberKnife is an image‐guided stereotactic radiosurgery system.^(^ ^15^ ^)^ By using a 6 MV linac, which is mounted on a robotic arm, CyberKnife can deliver multiple isocentric or non‐isocentric beams to a desired target. An integrated stereoscopic kV imaging system is used to monitor patient position throughout the course of treatment. By matching fiducial markers or bony anatomy from kV X‐ray images to DRRs generated from CT simulation, the resulting setup errors (three translations and three rotations) can be determined and compensated either automatically or manually by a therapist. Synchrony Respiratory Tracking system (Accuray Inc., Sunnyvale, CA) is an integrated tumor tracking component of the CyberKnife system^(^ ^16^ ^)^ that allows dynamic compensation for respiratory motion. The tumor position, which is determined through kV X‐ray visualization of adjacent fiducial markers, is correlated with the location of external markers using an adaptive model. By optically tracking the motion of external markers position, the adaptive model can predict the tumor position and guide the robot in tracking the tumor. The model can be updated during the treatment by subsequent kV imaging to validate the position of an internal reference. A recent report has shown that the Synchrony system can provide accurate tracking of lung tumors.^(^ ^17^ ^)^

Both linac‐based systems and the CyberKnife have been applied to SBRT treatment of lung cancer. However, there are no clear guidelines for patient selection between these two different modalities. The different characteristic of these two modalities, which may produce significantly different dose distributions, has not been addressed. A study conducted by Prevost et al.^(^ ^18^ ^)^ utilized the linear‐quadratic model to investigate biological aspects of CyberKnife SBRT and conventional 3D CRT for lung irradiation. There are significant uncertainties in the results, particularly as the linear quadratic model may require modification for large dose‐per‐fraction, as is the case in SBRT.^(^ ^19^ ^)^ Further, in the absence of 4D dose calculation, the GTV and PTV in this study are different between CyberKnife and 3D CRT due to patient respiratory motion. In an effort to address the different dosemetric characteristics of linac‐based SBRT and that of CyberKnife for lung tumor treatment, we present a matched comparison of treatment plans for both modalities. To minimize respiratory motion impact on dose calculation, a 4D dose calculation technique was used for linac‐based SBRT for comparison with Synchrony plans and delivery. Identical patient image and contour sets were used for dose calculation from both modalities, eliminating potential sources of bias due to variations in patient anatomy, normal tissue contouring and PTV definition. The results of this study may provide a general guideline for patient and treatment modality selection based on dosimetric, tumor control and normal tissue sparing considerations.

## II. MATERIALS AND METHODS

### A. Patients

Eight patients with lung tumors, previously treated using linac‐based SBRT, were included in this retrospective study. A published protocol^(^ ^3^ ^)^ was followed for all the patients treatment planning. Sixty Gy in three fractions was prescribed to cover 95% of target tumor. This study was approved by the institutional review board and conducted in accordance with institutional guidelines.

### B. 4D CT study

In this study and in our routine practice, all patients were placed in a stereotactic body frame, which has been described in literature.^(^ ^20^ ^)^ Abdominal compression^(^ ^10^ ^)^ was applied to all patients to reduce respiratory motion. A bellows system coupled to our 16‐slice Brilliance Big Bore CT scanner (Philips Healthcare, Andover, MD) was used to monitor the patients' respiratory motion for subsequent amplitude‐based sorting. After scanning, the 4D CT image data were sorted into 10 phases, such that the 0% respiratory phase corresponds to peak inhalation and the 50% respiratory phase corresponds to the peak exhalation. For each phase, a 3D CT volume image of the patient is created. These images allow physicians, physicists, and dosimetrists to localize a moving organ or target in each phase of breathing cycle.

### C. Linac‐based SBRT planning

Following CT scanning, three‐dimensional MIP images were created from the full 4D CT image sets. MIP images were subsequently exported to ${\text{Pinnacle}}^{3}$ 8.0m (Phillips Medical Systems, Cleveland, OH) treatment planning system for contouring. Lung tumors were contoured by a radiation oncologist on MIP images to create an ITV. The ${\text{PTV}}_{3D}$ used for linac‐based 3D conformal SBRT planning was obtained by adding a uniform three‐dimensional 5 mm margin to the ITV, which was used to compensate for setup uncertainties and residual respiratory motion not represented by 4D CT. Both lungs were automatically segmented using a threshold algorithm in Pinnacle; the ${\text{PTV}}_{3D}$ was subsequently subtracted for the lung volume. Other critical organs such as heart, esophagus and spinal cord were also contoured. 3D conformal SBRT plans were created for each target by experienced dosimetrists.

### D. 4D dose calculation

Clearly, the ${\text{PTV}}_{3D}$, which is expanded from the ITV, includes the patient respiratory motion information. Therefore, the resulting dose distribution does not accurately reflect the dose delivered to the target or surrounding critical organs in the presence of respiratory motion. In this study, a 4D dose calculation procedure was applied to calculate the 4D dose distribution without the impact of respiratory motion. A GTV and ${\text{PTV}}_{4D}$, which exclude the motion information, were contoured for dose evaluation. This procedure was performed in the following manner.

Because the MIP image and the 10 4D CT datasets are represented in the same DICOM coordinate space, the SBRT plan with all the beam information can be applied to each of the 10 4D CT datasets by mapping the DICOM coordinates. The dose is subsequently calculated on each of the 10 4D CT datasets. To calculate the cumulative radiation dose to moving anatomy, it is necessary to trace the deformation trajectory of each voxel during the respiratory cycle. An intensity‐based automatic deformable registration algorithm known as a “demons” algorithm^(^ ^21^ ^)^ was applied to track the respiratory motion of each voxel. In this study, the 50% respiratory phase of the 4D CT image set (corresponding to end exhalation) was chosen as the fixed reference, and the demons method was applied to match each of the other 4D CT respiratory phases to it. Similar work has been reported in the literature.^(^ ^22^ ^)^

Deformable registration builds the voxel‐to‐voxel correspondence between the moving image and fixed image. Given such information, the cumulative dose that the moving target receives during respiration can be calculated by using the following method. First, the radiation dose distribution is calculated on each respiratory phase for same beam configuration. Strictly, the probability density function derived from patient breathing files should be used to weigh the duration of each respiratory phase. In this study, this was simplified using equal respiration phases as an approximation. Therefore, the cumulative radiation dose $D_{i}$ of voxel *i* can be computed as:

(1) $$D_{i}=\sum_{j=1}^{N}D_{i\prime j}$$

where *N* is the number of respiratory phases, and $D_{i\prime j}$ is the dose of voxel *i′* at breathing phase *j*. The voxel *i′* corresponds to the voxel *i* at the 50% respiratory phase.

The GTV is contoured on the 50% phase of the 4D CT image set by experienced radiation oncologists. The ITV contour, which was drawn on MIP images, is used as a reference for GTV contouring to minimize normal tissue such as the chest wall from inclusion within the GTV. The ${\text{PTV}}_{4D}$ is then created by adding a uniform three dimensional 5 mm margin to the GTV. The 50% phase from the 4D CT, and the contours of the GTV and ${\text{PTV}}_{4D}$ are sent from the Pinnacle system to the multiplan system for CyberKnife treatment planning. Following this 4D dose calculation procedure, the respiratory motion impact is removed from the resulting dose distribution, which corresponds solely to the 50% phase of 4D CT images. Tumor volume and location, and lung volume, are listed for each patient in Table 1.

**Table 1:** Tumor and lung volume and tumor location.

*Patient*						*Tumor Position*	
	*GTV (cm^3^)*	${\mathrm{PTV}}_{4D}$ *(cm^3^)*	*ITV (cm^3^)*	${\mathrm{PTV}}_{3D}$ *(cm^3^)*	*Lung (cm^3^)*	*A/P*	*S/I*
1	2.2	15.5	3.1	18.3	4604	Anterior	Middle
2	9.7	34.3	10.3	36	2877	Anterior	Superior
3	7.6	24.8	9.7	33.7	2716	Middle	Superior
4	66.3	126	68.3	145.4	2653	Middle	Inferior
5	18.5	46.5	18.5	46.5	3122	Posterior	Middle
6	19.8	64.5	25.6	76.5	3713	Posterior	Inferior
7	2.4	12.7	3.1	14.6	2417	Posterior	Middle
8	10.5	31.6	17.1	43.5	3087	Posterior	Middle

### E. CyberKnife treatment planning

The Synchrony treatment planning procedure recommended by manufacturer and described in detail by Ozhasoglu et al.^(^ ^16^ ^)^ was used in this study. Since the CyberKnife Synchrony system can track the tumor during respiratory motion, the manufacturer recommends that the ${\text{GTV}}_{\text{Cyber}}$ is contoured on an end‐exhalation breath‐hold CT scan, which is similar to the 50% phase (maximum exhalation phase) of 4D CT images. The ${\text{PTV}}_{\text{Cyber}}$ for the CyberKnife treatment planning is defined by adding a uniform 5 mm margin to the ${\text{GTV}}_{\text{Cyber}}$ to compensate for residual setup error and respiratory motion, and for tumor deformation. In this manner, the recommended definition of the ${\text{GTV}}_{\text{Cyber}}$ and ${\text{PTV}}_{\text{Cyber}}$ for the CyberKnife Synchrony treatment planning is equivalent to the GTV and ${\text{PTV}}_{4D}$ defined for the 4D dose calculation in linac‐based SBRT. In this study, the GTV, ${\text{PTV}}_{4D}$ and 50% 4D CT images were transferred from Pinnacle to CyberKnife to ensure that the identical images and contour sets were used for both the 4D dose calculation in linac‐based SBRT and CyberKnife Synchrony treatment planning. For comparison purposes, a dose prescription of 60 Gy in three fractions designed to cover at least 95% of the ${\text{PTV}}_{4D}$ was used for both CyberKnife and linac plans.

### F. Study protocol

**Figure 1:**
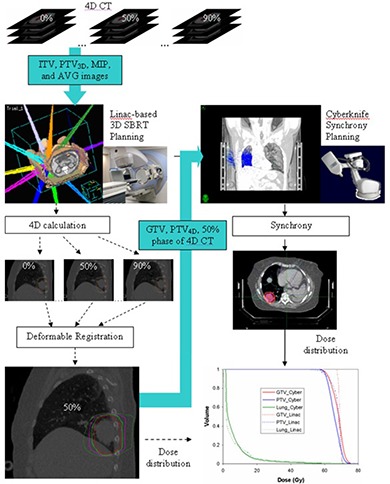
Diagram of study protocol. A 4D CT scanning is performed for all patients. A 3D conformal linac‐based SBRT plan is designed on ITV and corresponding ${\text{PTV}}_{3D}$. A deformable registration method is then applied to obtain the 4D cumulative dose distribution. GTV and ${\text{PTV}}_{4D}$ are contoured on the 50% phase of 4D CT images, which corresponds to the maximum exhalation. The 50% phase of 4D CT images and contour sets of GTV and ${\text{PTV}}_{4D}$ are sent to CyberKnife system for Synchrony planning.

Figure 1 shows the technical aspects of the study. A 4D CT scan is obtained for all patients. For linac‐based SBRT, a 3D conformal plan is designed based on the ITV and corresponding ${\text{PTV}}_{3D}$. A deformable registration method described in the previous section is then applied to obtain the 4D cumulative dose distribution. The GTV and ${\text{PTV}}_{4D}$ are contoured on the 50% phase of 4D CT images, which corresponds to maximum exhalation. The 50% phase volume, and the contours of the GTV and ${\text{PTV}}_{4D}$ are transferred to the CyberKnife system for Synchrony planning. Dose volume histograms (DVHs) for GTV, ${\text{PTV}}_{4D}$ and lung are calculated and compared between the linac‐based and CyberKnife SBRT plans.

A dose heterogeneity index (DHI) is introduced to numerically evaluate dose heterogeneity in target tumor. This index is defined as follows:

(2) $$\text{DHI}=100\times (D_{20}-D_{80})/D_{\text{prescription}}$$

where $D_{20}$ and $D_{80}$ represent the dose covering 20% and 80% of target volume, respectively, and $D_{\mathrm{prescription}}$ is the prescription dose. According to the definitions of $D_{20}$ and $D_{80}$, $D_{20}$ is always greater or equal to $D_{80}$. Therefore a lower index reflects a smaller difference between the doses covering 20% and 80% of the target volume, and indicates better dose homogeneity.

The equivalent path length method was applied in our current multiplan treatment planning system of CyberKnife for heterogeneous correction of lung treatment. This method corrects the density of lung without consideration of the heterogeneity with regard to lateral electron scatter. For comparison with CyberKnife plans, only primary photon fluence corrections were applied for lung heterogeneities in the Pinnacle plans.

## III. RESULTS & DISCUSSION

### A. Target coverage

**Figure 2:**
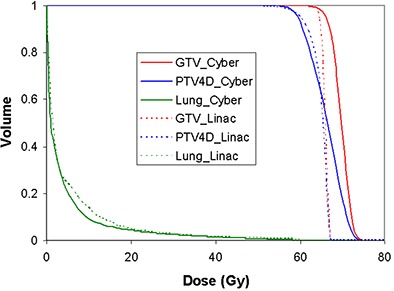
A typical DVH of GTV, ${\text{PTV}}_{4D}$ and lung for CyberKnife and linac‐based SBRT lung cancer treatment. The solid curves are the DVH for CyberKnife plan. The dash lines are the DVH for linac‐based SBRT plan.

Typical DVHs for the GTV, ${\text{PTV}}_{4D}$ and total lung (excluding the ${\text{PTV}}_{4D}$) are shown in Fig. 2. While both linac and CyberKnife can provide enough dose coverage for target tumor, the target dose distributions of the two modalities have different characteristics. Specifically, the dose to the GTV in the CyberKnife plan is more heterogeneous than that of linac‐based SBRT plans. Further investigation of the DHI in Table 2 shows that the average DHI of GTV is 8.71 (standard deviation of 1.87) for CyberKnife plans, which is statistically greater than 3.35 (standard deviation of 1.47) for linac‐based SBRT. The maximum dose to the GTV is also greater for CyberKnife than for linac‐based treatment. In contrast, there was no statistical difference in the DHI for ${\text{PTV}}_{4D}$ between these two modalities.

**Table 2:** DHI of PTV4D and GTV and maximum point dose to GTV.

*Patient*	*DHI for* ${\mathrm{PTV}}_{4D}$		*DHI for GTV*		*Maximum Point Dose to GTV (Gy)*	
	*CyberKnife*	*Linac*	*CyberKnife*	*Linac*	*CyberKnife*	*Linac*
1	11.94	8.19	10.09	2.31	78.2	70.3
2	7.86	9.91	9.01	3.51	72.4	72.2
3	12.13	4.82	5.93	2.38	74.3	66.9
4	9.84	7.46	11.28	5.43	75.2	71.3
5	9.08	12.98	6.24	5.84	73.3	73.2
6	14.1	5.48	8.36	2.17	77.1	68.1
7	9.98	14.14	8.62	2.61	73.3	73.2
8	10.77	6.64	10.13	2.53	76.2	70.2
mean±std	10.71±1.97	8.70±3.40	8.71±1.87	3.35±1.47	75.0±2.0	70.7±2.3

Difference in delivery characteristics between the CyberKnife and linac systems can explain the differences in tumor dose distributions. The CyberKnfie system does not have a flattening filter. Therefore, the CyberKnife dose profiles are less flat than that of linac systems for a given collimator size. Furthermore, to keep a balance between conformity and short treatment times, the cone size of CyberKnife was chosen as 60%~80% of smallest dimension of the ${\text{PTV}}_{4D}$. In contrast, the linac‐based system uses a beam aperture covering the entire ${\text{PTV}}_{3D}$, which encompasses the tumor motion. Therefore, the linac‐based plans employ a larger beam aperture than CyberKnife plans. Consequently, the linac‐based dose profiles are much flatter than those from the CyberKnife. Additionally, the number of beams and beam directions used by the CyberKnife (100~150 beams) are much greater than that used in linac‐based SBRT (7~10 beams). While this provides for high conformity in CyberKnife plans, it also increases the heterogeneity of resulting dose distribution. Conversely, the GTV dose distribution in linac‐based plans is more homogeneous than for CyberKnife plans.

For the ${\text{PTV}}_{4D}$, the dose heterogeneity in proximity to the tumor‐lung interface outweighs the impact of multiple unflattened beams used by CyberKnife. Accordingly, no statistical difference in the ${\text{PTV}}_{4D}$ DVIs was observed between both modalities.

The clinical relationship between tumor dose heterogeneity and tumor control in lung SBRT is poorly understood. While an earlier investigation suggested the dose heterogeneity generated by GammKnife radiosurgery may result in greater control of cranial tumors compared to linac‐based stereotactic radiosurgery,^(^ ^23^ ^)^ the conclusion has been disputed.^(^ ^24^ ^)^ Certainly those results cannot be generalized to apply to lung SBRT. Therefore, if the dose coverage of the PTV meets the dose constraints presented in the literature,^(^ ^3^ ^)^ no clinical difference between linac and CyberKnife SBRT can be predicted. Further investigation is expected to clarify what is the impact of the difference of dose heterogeneity found in our study to lung tumor control.

Table 3 shows the RTOG conformity index,^(^ ^25^ ^)^ defined as the ratio of the tissue volume receiving the prescription isodose or more to the tumor volume, for both modalities. It should be noted that although CyberKnife provided a more conformal dose to the target, the difference in conformity is due primarily to the smaller target volumes in CyberKnife treatment by robotic tumor tracking.

**Table 3:** Conformity index.

*Patient*	*CI*		*Tumor Position*
	*CyberKnife*	*Linac*	
1	1.22	2.14	Anterior
2	1.16	1.38	Anterior
3	1.09	1.89	Middle
4	1.21	1.62	Middle
5	1.15	1.27	Posterior
6	1.15	1.62	Posterior
7	1.21	1.44	Posterior
8	1.06	1.80	Posterior
mean±std	1.16±0.06	1.64±0.29	—

### B. Normal lung dose

Radiation‐induced pneumonitis is the most common complication of lung tumor treatment. The probability and severity of this complication is highly dependent on the lung dose and irradiated volume. Various models and dose parameters, based largely on the existing clinical experience with conventional fractions, have been used to predict the probability of complications.^(^ ^26^ ^)^ In estimating the likelihood of radiation‐induced lung toxicity, the result of model‐based predictions is highly correlated with DVH characteristics.^(^ ^27^ ^)^ Therefore, single point metrics such as $V_{20}$, which is the percentage volume receiving the dose of 20 Gy or higher, may be used as a single factor to estimate lung complication for conventional lung treatment. However, lung toxicity from a dose of 20 Gy given in few fractions is not predicted by conventional models or metrics^(^ ^19^ ^)^ and thus, it is unlikely that $V_{20}$ can adequately represent the possibilities of lung complication of SBRT treatment. Lung toxicity following SBRT treatment is a subject of much discussion and interest. A recent study by Stephans et al.^(^ ^28^ ^)^ has suggested that volume of normal lung within low dose region is associated with pulmonary function changes following SBRT. In the present study, we have used $V_{20}$ as a metric in the high dose region, and the minimum dose to $1000\;{\text{cm}}^{3}$ is used to characterize plan quality in low dose region.

**Figure 3:**
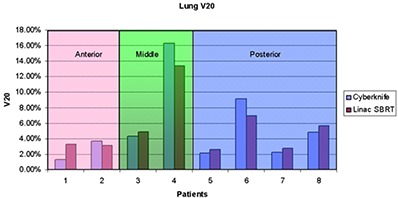
$V_{20}$ for CyberKnife and linac‐based SBRT treatment grouped with tumor location.

As shown in Table 4 and illustrated in Fig. 3, $V_{20}$ is very similar between both modalities for all of the patients. Further investigation shows that $V_{20}$ increases with increasing tumor volume for both modalities, and is strongly correlated with tumor volume and shape. It also indicates that both modalities can provide very similar conformal dose distributions around the target tumor.

**Table 4:** $V_{20}$ and minimal dose to $1000\;{\text{cm}}^{3}$ lung.

*Patient*	$V_{PTV4D}$	$V_{20}$		*Minimal Dose to* $1000\;cm^{3}$ *lung (Gy)*		*Tumor Position*
		*CyberKnife*	*Linac*	*CyberKnife*	*Linac*	
1	0.34%	1.34%	3.27%	2.55	5.77	Anterior
2	1.19%	3.67%	3.11%	2.86	4.28	Anterior
3	0.91%	4.31%	4.90%	2.07	2.35	Middle
4	4.75%	16.32%	13.37%	8.36	6.96	Middle
5	1.49%	2.16%	2.59%	3.76	2.30	Posterior
6	1.74%	9.11%	6.95%	7.86	4.10	Posterior
7	0.53%	2.21%	2.74%	2.75	2.91	Posterior
8	1.02%	4.83%	5.67%	5.85	4.00	Posterior
mean±std	1.5%±1.4%	5.5%±5%	5.3%±3.6%	4.2±2.6	3.8±1.6	—

**Figure 4:**
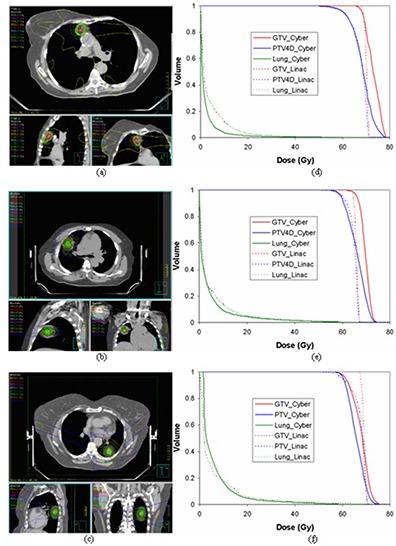
The impact of tumor location on DVH of CyberKnife and linac‐based SBRT lung cancer treatment: lung tumors are shown in (a), (b) and (c); (d), (e) and (f) are the corresponding DVHs, respectively.

**Figure 5:**
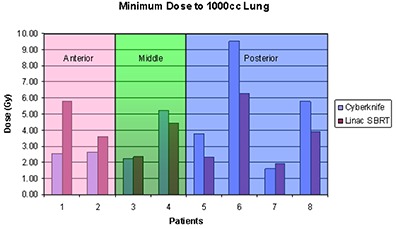
Minimal dose to $1000\;{\text{cm}}^{3}$ lung for CyberKnife and linac‐based SBRT treatment grouped with tumor location.

In the low dose region, both modalities also provided similar dose to 1000 cc lung for all of the patients (Table 4). However, tumor location is a more important factor in characterizing the lung dose distribution in this region. The present study shows that the lung dose from CyberKnife plans is more susceptible to the tumor location than that of linac‐based SBRT plan. As illustrated in Fig. 4, when the tumor attaches to the anterior chest wall, CyberKnife may deliver less dose to the lung than Linac‐based SBRT. When the tumor location is more posterior, the lung dose from CyberKnife plans/treatment can be significantly greater than that of Linac‐based SBRT plans/treatments. For patients with tumor attached to the posterior chest wall, the low dose volume from CyberKnife delivery is significantly greater than from linac‐based delivery. Figure 5 shows the minimum dose to $1000\;{\text{cm}}^{3}$ of lung for all the patients as a function of tumor location. The numerical results were given in Table 4.

These observations are due to several characteristics which are different between the two modalities. First, a typical CyberKnife SBRT treatment plan uses many more beams (typically more than 100 in our practice) than linac‐based SBRT treatment (7 to 10 beams). Second, limited by the accessible position of robotic arm, none of the CyberKnife beams can be delivered from underneath the patient. Therefore, while CyberKnife can deliver a more conformal dose to the tumor than Linac‐based SBRT and less dose to the lung when the tumor is attached to anterior chest wall, the opposite is the case for posterior‐located tumors.

Both modalities can provide a very conformal dose to the target tumor and spare the normal lung. Although the low dose to lung may be different between CyberKnife and linac‐based SBRT due to the tumor location, the lung dose is well below institutional constrain for all patients by both modalities. Therefore, applying different modalities in lung cancer treatment may not introduce dramatically different possibility of lung complication.

### C. Whole body dose

One of the results of the present study is that the total number of monitor units needed to deliver similar prescription dose is significantly different between these two modalities. The small aperture and unflattened beam profile limit the output of CyberKnife and increase the MU required to deliver similar dose to the target. For CyberKnife treatments, more than 25,000 monitor units were required, compared with 9000 to 15,000 monitor units required in linac‐based SBRT. As a result, CyberKnife delivery may result in a higher whole body dose to the patients. This is supported by the results from an interinstitution phantom study by treating prostate and brain tumors using linac‐based IMRT and CyberKnife.^(^ ^29^ ^)^

### D. Uncertainties for dose delivery

There are several uncertainties that may impact the actual dose distribution delivered to the patient. These uncertainties include setup error and residual respiratory motion which can not be represented by 4D CT. Generally, both setup error and residual respiratory motion can be treated as two independent random error sources. Therefore, these uncertainties can be added in quadrature to determine the total uncertainty.

In our study, abdominal compression, which usually can reduce the patient tumor motion to less than 1 cm, is applied to all patients treated with linac‐based SBRT. CT data are reconstructed with a slice thickness of 2 mm. Scans are acquired with an X‐ray tube rotation time of 0.5 s. These parameters are close to those reported in the study by Rietzel et al.^(^ ^30^ ^)^ who concluded that the accuracy of 4D CT images is one CT slice thickness. Although additional setup error may be introduced in the process of matching the CBCT to DRR generated by average image of 4D CT, the total uncertainties can be limited within few millimeters. Since the orthogonal X‐ray images matched to DRRs are used to set up patients for CyberKnife treatments and the Synchrony system is applied to tumor tracking throughout the entire treatment, similar level of uncertainties can be expected in CyberKnife treatment.

By applying the modern IGRT techniques, the patients' setup error and residual motion are limited to a few millimeters for both modalities. With the application of abdominal compression and the IGRT techniques described above, 5 millimeters GTV to ${\text{PTV}}_{4D}$ margin was adequate for providing acceptable compensation for setup error and residual breathing motion.

### E. Other treatment options for linac‐based systems

Treatment techniques such as respiratory gating and IMRT may further reduce the irradiated volume of normal lung in linac‐based SBRT. Therefore, improved dosimetric result for linac‐based SBRT treatment planning can be expected by applying these techniques. However, careful consideration should be made before applying these techniques.

The application of respiratory gating would increase delivery time by a factor of 3 or more over that of conventional delivery, depending on duty cycle of gating, the beam on and off effects, and patient compliance. Additional imaging may also be required during treatment to verify/confirm target location, further extending treatment time. Increases in delivery time may also negatively impact patient's comfort, increase chances of patient movement and decrease patient throughput. A recent study of gating treatment for SBRT also concluded that if the tumor motion less than 15 mm, free breathing treatment is preferable.^(^ ^31^ ^)^

Although IMRT can improve dose conformity of linac‐based treatment planning, respiratory motion may present considerable dose variation if IMRT is used.^(^ ^32^ ^)^ Although this effect may average/blur out in treatments with many factions, for SBRT treatment with only few fractions, it may become significant. Treatment time will also be increased by applying IMRT technique in SBRT treatment.

## IV. CONCLUSIONS

Both Linac and CyberKnife SBRT systems can provide adequate dose coverage for target tumor. While the CyberKnife may deliver less lung dose than linac‐based systems for tumors close to the anterior chest wall, the converse is true for tumors located posteriorally. The magnitude of differences in lung dose between both modalities due to tumor position is relatively small. CyberKnife requires more MU to deliver similar target prescription to the tumor than linac SBRT systems. The results of this study may provide a general guideline for patient and treatment modality selection based on dosimetric, tumor control and normal tissue sparing considerations.
